# Marrow Fat-Secreted Factors as Biomarkers for Osteoporosis

**DOI:** 10.1007/s11914-019-00550-w

**Published:** 2019-11-16

**Authors:** Markus Herrmann

**Affiliations:** Clinical Institute of Medical and Chemical Laboratory Diagnostics, Medical University of Graz, Auenbruggerplatz 15/1, 8036 Graz, Austria

**Keywords:** Bone marrow fat, Adipocytes, Leptin, Adiponectin, Fatty acids

## Abstract

**Purpose of Review:**

The age-related accumulation of bone marrow adipose tissue (BMAT) negatively impacts bone metabolism and hematopoiesis. This review provides an overview about BMAT-secreted factors as biomarkers for BMAT accumulation and osteoporosis risk.

**Recent Findings:**

The adipokines leptin and adiponectin are regulators of BMAT. It remains to be clarified if locally produced adipokines substantially contribute to their peripheral serum levels and if they influence bone metabolism beyond that of extraosseous adipokine production. Existing data also suggests that BMAT disturbs bone metabolism primarily through palmitate-mediated toxic effects on osteoblasts and osteocytes, including dysregulated autophagy and apoptosis.

**Summary:**

BMAT-secreted factors are important modulators of bone metabolism. However, the majority of our understanding about MAT-secreted factors and their paracrine and endocrine effects is derived from in vitro studies and animal experiments. Therefore, more research is needed before BMAT-secreted biomarkers can be applied in medical practice.

## Introduction

For many decades, adipose tissue has been considered as an energy store where excess carbohydrates and fatty acids are stored in the form of lipids. In recent years, it has emerged that fat tissue has a wide range of other paracrine, endocrine, and immune functions [1–3]. Furthermore, there are different types of adipose tissue that are located at distinct anatomical locations [1–3]. White adipose tissue (WAT), for example, represents the vast majority of all body fat in humans and is primarily located in abdominal and subcutaneous depots [4]. WAT has the capacity to adapt and expand in response to excess energy intake through adipocyte hypertrophy and/or recruitment and proliferation of precursor cells in combination with vascular and extracellular matrix remodeling. In contrast, brown adipose tissue (BAT) accounts for approximately 10% and is predominantly organized in smaller cervical, supraclavicular, and paravertebral depots. Despite the fact that the adipocytes of WAT and BAT are key players in energy metabolism, their morphology and function differ substantially [2]. WAT is mainly composed of unilocular cells, with a high capacity to store triglycerides, whereas BAT contains multilocular adipocytes, which are rich in mitochondria [1, 2]. Brown adipocytes are able to burn high amounts of energy through the uncoupling protein-1. They can take up large amounts of glucose and have a high oxidative capacity. The resulting heat production is a physiological response to cold stress.

Besides the well characterized subcutaneous and visceral adipose tissue, substantial amounts of fat are also present in the bone marrow. However, the metabolic role of bone marrow adipose tissue (BMAT) has long been ignored. Throughout the lifetime of a human, hematopoietic bone marrow (BM) is continuously replaced by adipocytes [5]. At birth, the cavities of trabecular bones are predominantly filled with blood-forming red bone marrow [6]. Already by age 25, 70% of BM is replaced by BMAT [7] and this centripetal accumulation of adipocytes continues until the end of life [8]. A closer look at BMAT has revealed that the adipocytes of MAT are distinct from those in WAT and BAT [2]. A lacking correlation between the amount of BMAT and total body fat supports this concept [9–12]. Only in obese premenopausal women visceral adiposity appears to correlate with vertebral BMAT [13]. Moreover, BMAT is not catabolized during acute starvation but increases during conditions of prolonged caloric restriction, including anorexia nervosa [14–19].

## Characteristics of Bone Marrow Adipocytes

Bone marrow adipocytes (BMAs) are derived from bone marrow mesenchymal stromal cells and account for 10% of total adipose tissue mass in humans. Histologically, bone marrow adipocytes accumulate lipid in a unilocular droplet that replaces nucleus and cytoplasm gradually [16, 20, 21]. They seem to be a heterogeneous population with distinct metabolisms, lipid compositions, secretory properties and functional responses, depending on their location in the bone marrow [22]. For example, in the tibia of mice, BMAs strongly express the typical BAT markers deiodinase (Dio) 2, peroxisome proliferator–activated receptor gamma coactivator (Pgc) 1α, and transcription factor positive regulatory domain-containing (PRDM) 16, but not UCP-1. In contrast, in vertebral BMAs, UCP-1 is present. Furthermore, it appears that there are substantial differences among species and between genders. As BMAs represent characteristics of brown and white adipocytes, they are referred to as beige adipocytes [23, 24]. Mature beige adipocytes possess multiple lipid droplets, but the number is typically lower than in white adipocytes. Moreover, the density of mitochondria is also lower. All BMAs express PPAR-γ, a ubiquitous nuclear receptor in adipocytes that regulates fatty acid storage and glucose metabolism. The primary function of BMAs is the storage of lipids, but upon stimulation they can transdifferentiate into a brown-like phenotype with thermogenic function [25, 26]. It should be kept in mind that most studies characterized brown and beige adipocytes through gene expression signatures that have been identified in mice, but commutability of these markers in humans is unclear.

BMAT has long been considered relatively inert, but recent evidence suggests that it possesses important metabolic, paracrine, and endocrine functions. In fact, BMAs secrete numerous signaling molecules including adipokines, FOXC2, Wnt, insulin-like growth factor, BMP, MCP-1, uncoupling protein-1, preadipocyte factor-1, and inflammatory markers [23, 24]. Furthermore, an expansion of BMAT during aging and obesity negatively impacts bone metabolism [27, 28] and hematopoiesis [29]. Initially, it was believed that these effects are mainly due to a redirection of precursor cells towards the adipogenic lineage. More recent studies suggest that there are also other mechanisms involved that are based on molecules secreted by BMAs. The purpose of this review is to provide an overview about these molecules and their role in bone metabolism.

## BMAT-Secreted Molecules

There is increasing evidence that BMAs exert detrimental effects on bone metabolism and hematopoietic bone marrow through a diverse range of secreted factors that include adipokines, fatty acids, extracellular vesicles, pro-inflammatory cytokines, and other regulatory proteins such as receptor activator of nuclear factor kappa B ligand (RANKL) and stem cell factor (SCF) [30]. Comparative studies between different types of adipose tissue suggest that synthesis and secretion of these factors are differentially regulated. In the following paragraphs, existing knowledge about MAT-secreted factors and their regulation is summarized.

## Leptin

Leptin is a 146 amino acid proteohormone that reduces fat mass and suppresses hunger by counteracting the hypothalamic effects of neuropeptide Y and anandamide, two potent hunger promotors [31, 32]. Furthermore, it stimulates the synthesis of α-MSH, a hunger suppressant [33]. Leptin is also known as “energy expenditure hormone” because it downregulates energy storage. The hormone is predominantly produced by adipocytes and enterocytes in the small intestine and acts on cell surface receptors in the arcuate nucleus of the hypothalamus [34]. Smaller amounts of leptin can also be synthesized in placenta, breast, stomach, bone marrow, hypothalamus and the pituitary gland [35–37]. Similarly, leptin receptors have also been found in many tissue types outside the brain, such as bone, muscle, blood vessels, kidney, bowel, placenta, pancreas, and immune cells [38, 39].

In humans, serum leptin concentrations are modified by many factors including fasting [40–42], sleep deprivation [43, 44], and emotional stress [45]. Regular physical exercises reduce circulating leptin chronically [46–48]. Therapeutic drugs, such as insulin or corticosteroids, can also alter serum leptin [49, 50].

In bone, leptin receptors are expressed on osteoblasts, osteoclasts, and BMSCs [51]. Hence, it is not surprising that leptin is a regulator of bone mass through direct and indirect effects on bone metabolism [52]. When peripherally acting on bone, leptin acts as a positive regulator of osteogenesis by increasing bone mineral density and bone formation rate while decreasing marrow adipogenesis [53]. The hormone enhances osteoblastic proliferation and differentiation while inhibiting adipogenic differentiation from marrow mesenchymal stromal cells [54]. Furthermore, it decreases cancellous bone, but increases cortical bone [55, 56]. Hamrick et al. [57] suggested that this represents an adaptation to increase bone size and bone resistance in obese individuals. Binding of leptin to leptin receptors on BMSCs suppresses adipocyte formation and promotes osteoblastic differentiation. Moreover, leptin inhibits osteoclast formation through an increase of osteoprotegerin (OPG), which is associated with a reduction of RANKL. Modulatory effects on fibroblast growth factor 23 and osteocalcin have also been reported [58].

Binding of leptin to central receptors in the hypothalamus instead induces indirect effects on bone through an activation of the sympathetic nervous system. This sympathetic activation results in a release of noradrenalin, which binds to the β2-adrenergic receptor on osteoblasts and inhibits bone formation [59]. It also increases RANKL expression and promotes the differentiation of osteoclasts.

In blood, leptin circulates in free and protein bound forms [60]. Blood concentrations vary exponentially with fat mass and differ between males and females [60–62]. The latter have markedly higher serum concentrations. Furthermore, leptin is subject to a circadian rhythm with highest concentrations between midnight and early morning, perhaps suppressing appetite during the night [63]. Meal-timing seems to modify the diurnal rhythm of blood leptin [62].

The expression leptin in BMAs has first been reported 20 years ago by Laharrague et al. [64], and others confirmed this finding later on [65–69]. The relative amount of leptin produced by BMAs seems to differ from WAT and between species [30]. Also, the contribution of MAT-derived leptin to peripheral serum leptin concentration is poorly studied. Reducing marrow adiposity in rats by sympathetic nervous blockade results in a substantial reduction of serum leptin concentration [70••]. Conversely, the expansion of MAT in mice after the induction of aplastic anemia leads to a marked increase of peripheral serum leptin by approximately 50% [71]. It remains to be clarified if local leptin production influences bone metabolism beyond that of leptin production outside the bone marrow cavities.

Measurement of leptin in serum and plasma is typically performed by immunoassays [72]. Initially, most studies used radioimmunoassay (RIA), but nowadays, other forms of immunoassay (e.g., enzyme-linked immunoassay (ELISA), chemiluminescence immunoassay (CLIA)) are increasingly used. Although there are only a very few assay comparison studies available, results suggest that the comparability of results is rather good and the sensitivity of most assays sufficient for clinical practice [73]. In humans, serum leptin concentrations are modified by many factors including fasting [40–42], sleep deprivation [43, 44], and emotional stress [45]. Regular physical exercises reduces circulating leptin chronically [46–48]. Therapeutic drugs can also alter serum leptin [49, 50]. In summary, leptin is an important indicator of marrow adiposity. However, multiple influencing factors and the overlaying effect of leptin secreted by WAT and BAT outside the bone marrow cavities strongly limit the utility of serum measurements for the assessment of MAT in clinical practice.

## Adiponectin

Adiponectin is another adipocyte-derived protein hormone that is involved in glucose homoeostasis and fatty acid oxidation [74]. It is composed by 247 amino, which are organized in four distinct regions. The hormone is secreted into the blood stream where it circulates in relative abundance compared with other hormones. It accounts for approximately 0.01% of all plasma protein at around 5–10 μg/mL (mg/L). Adiponectin automatically self-associates into homotrimer, hexamers, and dodecamers. Females have an increased proportion of these high molecular weight forms, which are believed to be biologically most active in glucose metabolism [75, 76].

Despite several reports that adiponectin is inversely correlated with body mass index [77], a later meta-analysis did not confirm this association in healthy adults [78]. In contrast to leptin, adiponectin is decreased in obese individuals and type 2 diabetics. Conversely, circulating adiponectin concentrations increase during caloric restriction in animals and humans, such as in patients with anorexia nervosa [79, 80]. This suggests a substantial contribution of MAT to circulating adiponectin levels because MAT expands during caloric restriction [81]. Adiponectin appears to impair adipocyte differentiation and to increase energy expenditure associated with mitochondrial uncoupling [82]. Consequently, it prevents or improves metabolic derangements, such as in type 2 diabetes [77] obesity, atherosclerosis [74], nonalcoholic fatty liver disease (NAFLD), and metabolic syndrome [83]. Similar to leptin, adiponectin exerts some of its weight-reduction effects via the brain [84] and both adipokines can act synergistically [85]. For example, together with leptin, adiponectin can completely reverse insulin resistance in mice [85].

Adiponectin is expressed and secreted by MSC-derived adipocytes [66, 86–88]. In BMAs, the expression of adiponectin seems to be lower than in peripheral adipocytes [89, 90]. However, most of these studies worked with isolated BM adipocytes. Analyzing intact MAT instead, Cawthorn et al. found a greater adiponectin secretion from MAT than from WAT in anorexia nervosa and during cancer therapy [81, 91].

Similar to leptin, the measurement of adiponectin in serum or plasma is performed by RIA or ELISA. Despite good correlation between RIA and ELISA methods, there is a lack of agreement between different methods preventing direct comparison of results [92]. In addition, there is neither an international reference standard nor a reference method available. Meanwhile, there are also ELISAs for the specific measurement of high molecular weight adiponectin, but their practical utility is insufficiently understood.

Adiponectin receptors have been shown to be present on osteoblast and osteoclasts [93, 94]. According to the data from the Health, Aging, and Body Composition study, adiponectin levels, but not leptin, were found to be associated with fracture risk among 3075 men and women, aged 70–79, suggesting that elevated adiponectin may be a risk factor for fractures independent of bone mineral density and body composition [95]. Altered adiponectin secretion with increased pro-inflammatory cytokines has been found to be associated with increased bone loss at the hip in overweight women [96]. In contrast to leptin, adiponectin decrease the sympathetic tone, which results in an increase in bone mass, thus preventing age-related bone loss [97]. The average number of BMAs in distal femur of adiponectin-knockout male mice at 37 weeks of age was significantly higher than in wild type mice, but no difference was found in the total adipocyte area of the two groups at all ages. These APN-KO mice had reduced cortical area fraction and average cortical thickness in all the age groups [98]. In conclusion, our current understanding of the impact of bone marrow adipocyte-derived adiponectin on bone metabolism and adiponectin serum concentration is limited. Multiple paracrine and endocrine mechanisms seem to mediate the role of adiponectin in bone and energy metabolism. These effects include the modulation of the sympathetic tone and insulin sensitivity [99].

## Extracellular Vesicles

Similar to many other cell types, adipocytes are capable of releasing extracellular vesicles (EVs), which carry proteins, nucleic acids, lipids, metabolites, and even organelles from the parent cell. These vesicles are surrounded by a lipid bilayer and vary in size between 20 nm and 10 μm. The content of EVs can be internalized by other cells and thus influence the function of recipient cells. Because of these characteristics, the diagnostic and therapeutic utility of EVs has been studied extensively [100–102]. Different types of EVs have been defined based on their size, origin, and function, e.g., microvesicles, microparticles, exosomes, and apoptotic bodies [103]. The overlapping and often contradictory definitions of different EV subtypes have led to the term “extracellular vesicle” as the most widely accepted scientific consensus. Similar to many other cell types, MSCs [104–108] and adipocytes [109–111] are capable of releasing EVs. Considering the proximity of BMAs and osteoblasts, the transfer of adipocyte-specific transcripts by EVs, including PPAR-γ, leptin, and adiponectin is an interesting concept. Such a cross talk could modulate the differentiation of MSCs into either adipocytes or osteoblasts [109]. In a co-culture model of human MSC-derived adipocytes and osteoblasts, Martin et al. showed that the latter cell type produces lesser amounts of osteogenic markers (e.g., osteocalcin) but expresses typical adipogenic genes [112]. A few years later, the same group reported the release of bone marrow adipocyte-derived EVs that contain adipocyte-specific transcripts, such as PPAR-γ, CEBPα, CEBPδ leptin, adiponectin, and anti-osteoblastic miRNAs miR-30c, miR-31, miR-125a, miR-125b, and miR-138. Furthermore, it appears that in this co-culture model EVs are involved in the inhibition of osteoblast activity. However, at present, this observation has not been confirmed by others. Furthermore, in vivo evidence is lacking. Therefore, additional evidence is needed to improve our knowledge about bone marrow adipocyte-derived EVs and their function.

## Lipids and Fatty Acids

BMAs represent a heterogeneous population. More than 40 years ago, Tavassoli identified different subpopulation based on their histochemical properties [113]. Upon performic acid-Schiff (PFAS) staining, adipocytes within the red marrow developed the typical red/purple color whereas adipocytes in the yellow marrow are remained unstained. PFAS staining exploits the oxidation of performic acid to free acetaldehyde in the presence of unsaturated fatty acids. Subsequent treatment with Schiff’s reagent leads to the production of a red/purple color [114]. In addition, PFAS-stained adipocytes in the red marrow disappeared in response to hemolysis, while nonstained adipocytes in yellow marrow remained unaffected [115]. Based on this observation, it was concluded that adipocytes in the red marrow contain predominantly unsaturated fatty acids. Recently, Scheller et al. confirmed the existence of region-specific variation in the properties of BMAs [116]. Furthermore, it has been shown that in early life, BMAs develop predominantly within the yellow marrow of the distal skeleton and remain there throughout life. In contrast to Tavassoli’s results, these constitutive BMAs have been found to contain predominantly unsaturated lipids. Red BMAs occur later in life and store primarily saturated lipids. These cells have been named regulated bone marrow adipocytes and are predominantly found in lumbar/thoracic vertebrae, proximal limbs, and ribs.

BMAs are secretory cells that exert paracrine and endocrine effects through the release of a broad range of molecules including adiponectin, leptin, RANKL, and lipids [30]. The release of free fatty acids from bone marrow into the blood stream has already been shown 35 years ago by vascular catheterization experiments [117]. Liberating stored triacylglycerols from adipocytes requires the stimulation of lipolysis, generally mediated by catecholamine-based activation of β-adrenergic receptors [118, 119]. Stimulating BMAs with isoproterenol increases the release of free fatty acid presumably through induction of lipolysis [117]. For example, cold exposure leads to an elevated sympathetic tone and concurrent BMAT depletion [116]. However, lipid hydrolysis induced by ß-adrenergic stimulation is substantially less in BMAT than in WAT. This relative resistance of BMAs to β-adrenergic stimulation separates them from other regulators of energy metabolism. The analysis of free fatty acids in bone marrow stem cell–derived adipocytes and adult WAT adipocytes revealed substantially different patterns [120••]. WAT adipocytes contain significant percentages of saturated and monounsaturated FFAs, including palmitic acid (C16:0), stearic acid (C18:0), and oleic acid (C18:1); some polyunsaturated FFAs, such as linoleic acid (C18:2), a small percentage of arachidonic acid (C20:4), and very little linolenic acid (C18:3). In comparison, bone marrow stem cell–derived adipocytes contain a comparable percentage of palmitic acid, more stearic and arachidonic acids, less oleic acid, almost no linoleic acid, and no detectable linolenic acid. Furthermore, they lack essential FFAs that may directly affect signaling related to their lipolysis/lipogenesis functions.

Bone marrow adiposity is believed to cause high levels of fatty acids within the bone marrow milieu, which has toxic effects on osteoblasts and osteocytes [121]. A series of in vitro studies demonstrated detrimental effects of palmitic acid on bone cells [122••, 123]. The intrinsic mechanisms of palmitic acid–induced lipotoxicity in osteoblasts and osteocytes include dysregulated autophagy and apoptosis. In another study, Casado-Díaz et al. [124] found that omega-6 arachidonic fatty acid, but not the omega-3 fatty acids, inhibits osteoblastogenesis and induces adipogenesis of human mesenchymal stem cells.

Although existing data suggests that bone marrow adiposity disturbs bone metabolism through palmitate-mediated toxic effects on osteoblasts and osteocytes, important pieces of information are currently lacking. It has not been shown that bone marrow adiposity leads to higher free fatty acid concentrations in the extracellular space. Furthermore, it is not clear if palmitate levels increase with accumulating bone marrow fat. Free fatty acids are typically measured by gas chromatography mass spectrometry (GCMS). This technology is not trivial and requires advanced expertise. Last but not least, mechanistic studies have exclusively been performed in cell culture studies. Hence, further studies are needed to confirm the relevance of these mechanisms in humans.

## Inflammatory Factors

Obesity is associated with chronic low-grade inflammation, impaired innate and adaptive immune responses [125, 126]. Similar to white adipocytes, BMAs can produce pro-inflammatory cytokines, such as TNF-α, IL-6, and IL-1 [64, 90]. Gene expression studies in mice suggest that there is a higher expression of TNF-α and IL-6 in BMAs than in white adipocytes from different anatomical locations [90]. However, in distinct metabolic situation, this pattern can be different. In mice fed with a high-fat diet, mRNA-levels of pro-inflammatory cytokines have been shown to be increased in visceral adipocytes, but decreased in BMAs [127]. Taken together, these observations suggest a differential regulation of the inflammatory response in different types of adipocytes upon stimulation. Until today, the regulation of cytokine production by human BMAs has been poorly studied. Laharrague et al. [64] have found only traces of IL-1ß and TNF-α in cultured human BMAs. At the same time, these cells produced significant amounts of IL-6. Another study showed that BMAs also express CXCL12, IL-8, colony-stimulating factor 3, and leukemia inhibitory factor [128]. Just recently, Mattiucci et al. reported a higher mRNA expression of pro-inflammatory cytokines in human MAT than in adipose tissue from the thigh [129]. Moreover, after 3 days of culture, IL-6, IL-8, and TNF-α were increased in the cell culture medium. The authors concluded that through the secretion of high levels of pro-inflammatory cytokines BMAs contribute to inflammation and impairment of plasma cell function in the bone marrow. Furthermore, they suggested MAT has more immune regulatory functions than WAT.

Although many open questions remain, pro-inflammatory cytokines are likely to mediate at least partially the detrimental effects of bone marrow adiposity [130, 131].

## Miscellaneous

Interestingly, BMAs are also capable of synthesizing RANKL. This has been shown in genetically modified mice where the PTH/PTHrP receptor was deleted in mesenchymal stem cells [132]. RANKL levels were also elevated in bone marrow supernatant and serum, but undetectable in other adipose depots. Also, human BMAs have been reported to express RANKL upon stimulation with dexamethasone [133]. In line with this observation, the RANKL/osteoprotegerin (OPG) mRNA ratio also increased. The same group also demonstrated that primary human BMAs support TNF-α-induced osteoclast differentiation through RANKL expression [134].

Plasminogen activator inhibitor-1 (PAI-1) is an inhibitor of fibrinolysis that is secreted from visceral and subcutaneous adipocytes. Stimulating primary human bone marrow adipocyte with dexamethasone strongly increases the expression and secretion of PAI-1 [135]. This effect can be attenuated by simvastatin. It has been speculated that simvastatin might exhibit preventive effects against steroid-induced osteonecrosis of the femoral head by suppressing PAI-1 secretion

In addition to the molecules described before, BMAs express and secrete a variety of other factors, such as SCF or dipeptidyl peptidase (DPP) 4 [29, 136]. The regulation and function of these factors are insufficiently understood. DPP4 is a protease that is involved in glucose homeostasis [137]. With increasing age, DPP4 is increased in distal tibiae bone marrow, which suggests that MAT modulates glucose metabolism through secreting DDP4 [29]. The secretion of SCF instead appears to be an important component that promotes hematopoietic regeneration in the hematopoietic stem cell niche [136].

## Conclusion

In recent years, it has become clear that MAT is much more than a simple energy store. It has distinct morphologic and biochemical features. BMAs secrete a broad range of factors that interact with the surrounding bone tissue and hematopoietic bone marrow. These factors include adipokines, fatty acids, extracellular vesicles, and pro-inflammatory cytokines. In addition, MAT has unique functions in the regulation energy metabolism. However, the majority of our understanding about MAT-secreted factors and their paracrine and endocrine effects are derived from in vitro studies and animal experiments. Therefore, it remains to be seen if these concepts hold true in humans. The measurement of most MAT secreted factors is rather difficult and requires advanced technical expertise. Furthermore, it is very difficult to distinguish MAT-derived molecules in peripheral or bone marrow blood from those produced elsewhere in the body. In summary, there are lots of open questions that should be addressed by future studies.
